# Left-sided cholecystitis in a patient with situs inversus totalis, complicated by portal venous malformations

**DOI:** 10.1093/jscr/rjad627

**Published:** 2023-11-20

**Authors:** Rishabh Suvarna, Ankit Gupta, Nasira Amtul

**Affiliations:** School of Medicine, Worsley Building, University of Leeds, Woodhouse, Leeds LS2 9JT, United Kingdom; School of Medicine, Worsley Building, University of Leeds, Woodhouse, Leeds LS2 9JT, United Kingdom; Leeds Institute of Emergency General Surgery, St James's University Hospital, Beckett Street, Leeds LS9 7TF, United Kingdom

**Keywords:** situs inversus totalis, cholecystitis, general surgery

## Abstract

Situs inversus totalis (SIT) is a rare autosomal recessive anomaly in which the thoracoabdominal viscerae are laterally transposed, introducing unique challenges in surgical scenarios. Only a few reports have demonstrated the treatment of cholecystitis in situs inversus, much less so in the context of portal vascular anomalies. We present the case of a 41-year-old female presenting to the emergency department with right upper quadrant pain, and subsequently found to have left-sided cholecystitis complicated by SIT with portal venous malformations on magnetic resonance cholangiopancreatography and abdominal computed tomography. Initially, she was referred for open cholecystectomy however due to the lack of symptoms and the presence of a tortuous recanalized portal vein presenting multiple thrombotic complications, an expectant approach was adopted. Thus, imaging remains the gold-standard to diagnose SIT and consideration of all congenital risk factors to cholecystectomy is crucial to avoid post-operative complications.

## Introduction

Situs inversus totalis (SIT) is a rare disorder occurring in ~1 in 5000 to 1 in 10 000 individuals and commonly inherited in an autosomal recessive pattern [1], involving a lateral inversion of the thoracoabdominal viscera. This was first described by Aristotle in animals [2], and first confirmed by Vehsemeyer through x-ray imaging [3], becoming the gold-standard to diagnose SIT. However, many surgeons lack the practical experience in dealing with such cases with many encountering it seldomly in their lifetimes [4]. Consequently, SIT can delay the diagnosis and treatment of cholecystitis due to surgical challenges in accessing the gallbladder. Since Chaouch et al.’s systematic review in 2021, documenting 93 case reports available in the literature [5], few additional cases on cholecystitis treatment in SIT patients have been reported, much less so in the context of portal hypertension.

We herein present the unusual case of a middle-aged female investigated for left-sided cholecystitis secondary to SIT, complicated by portal venous malformations.

## Case report

A 41-year-old female presented to the emergency department with acute cholecystitis and a long history of symptomatic gallstones. She was hemodynamically stable but had a raised white blood cell count and was pyrexic, suggestive of infection. Upon general inspection, the patient did not suffer from jaundice, fever, or emesis. However, she felt nauseous with epigastric and left upper quadrant pain, exacerbated post-prandially. On examination, the abdomen was soft and non-tender, presenting with a positive Murphy’s sign and a palpable liver on the left. The patient produced normal stool and clear urine.

Past medical history confirmed complete situs inversus with dextrocardia and a left-sided liver, and portal hypertension secondary to portal venous malformations, that required oesophageal variceal banding in 2015. The patient also had a mechanical aortic valve replacement in 2013. Thus, this gave an overall impression of biliary colic, reinforced by deranged liver function tests and ultrasound demonstrating a dilated common bile duct of 9 mm with gallstones.

**Figure 1:**
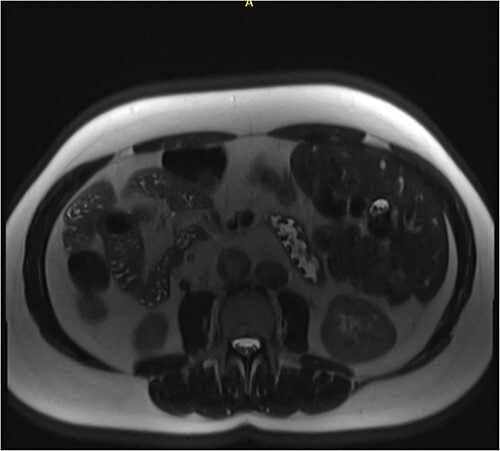
Axial MRCP scan demonstrating small stones in a contracted gallbladder.

**Figure 2:**
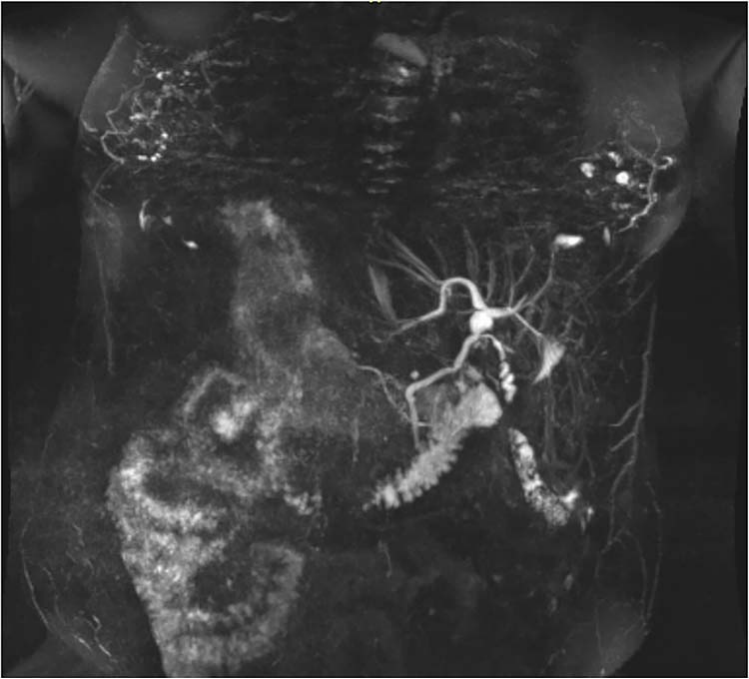
Coronal MRCP scan demonstrating a 10 mm area of saccular duct dilatation at the hilar confluence. Large, dilated vessels/varices surrounding the gallbladder fossa can also be appreciated.

Following examination, a magnetic resonance cholangiopancreatography (MRCP) test was requested to exclude choledocholithiasis. 24 hours later, this confirmed gallstones, with 10 mm saccular dilatations near the hilar confluence (Figs 1 and 2). This dilatation was non-progressive according to previous scans, with unaffected pancreatic and intrahepatic ducts. Dilated portal vessels and varices were discovered to be directly communicating with the gallbladder fossa, suggestive of portal venous communication.

Being largely asymptomatic, she was conservatively managed by the general surgery team and was referred to the liver surgeons for consultation regarding an open cholecystectomy, with abdominal computed tomography (CT) scans requested for assessing portal vein collaterals around the gallbladder.

Follow-up with a congenital cardiac consultant confirmed that there would be no major cardiac risks associated with cholecystectomy. However, the main risk identified by abdominal CT scans was the tortuous recanalized portal vein circumscribing the gallbladder and providing main portal inflow to the liver, presenting a high risk of bleeding, portal venous thrombosis, or worsened portal hypertension. Given the risks of surgery and minimal symptoms, the patient opted for a ‘watch and wait’ approach.

## Discussion

The heterogenous nature of SIT poses significant challenges to characterize it with a standard clinical vignette. Such abnormalities can either present as situs ambiguus, abnormal organ positioning in isolation, or situs inversus, a lateral reversal of organ systems. With >100 genes involved, it can manifest with partial reversal of thoracic or abdominal organs as situs inversus partialis, or complete reversal as SIT. SIT is associated with other congenital abnormalities such as congenital heart disease, biliary atresia, and vascular anomalies, all of which should be considered prior to surgery [6].

While current literature suggests SIT itself does not increase the incidence of cholecystitis, it often causes diagnostic confusion, as misdiagnoses for gastritis, pancreatitis are common when assessing left upper quadrant pain. Approximately 30% of SIT patients with cholecystitis can also present with epigastric pain and 10% can present with right-sided positive Murphy’s sign, illustrating the possible lack of transposition of the central nervous system [7]. Thus, a strong index of clinical suspicion, along with greater awareness of SIT-related anatomical anomalies and greater utilization of high-resolution imaging modalities are necessary to accurately confirm SIT and for careful surgical planning.

A lack of timely diagnoses with imaging can delay surgical time, requiring more time to adjust to the unusual anatomy and rearrange equipment accordingly. While laparoscopic cholecystectomies are deemed feasible and safe to perform in SIT, it requires right-handed surgeons to be comfortable with utilizing their left-hand to navigate left-sided ports or able to mirror their manoeuvres. Ergonomic challenges such as hand crossovers, trunk hyperflexion during Calot’s triangle dissection, and Hartmann’s Pouch retraction contribute the largest proportion of additional operative time [8] and often require two surgeons to individually perform each procedure safely and efficiently [9].

Limited literature exists on the relationship between SIT and the incidence of portal vascular malformations and portal hypertension [10], with mixed results being reported. To our knowledge, there are some case reports detailing abnormal portal vein positioning [11, 12], but none describing recanalized portal veins that circumscribe the gallbladder as is in our case. This can be a major contra-indication for laparoscopic cholecystectomies and hence warrants further research, as this can severely increase the risk of iatrogenic injury. In such cases, conducting a thorough pre-surgical screening of patients with SIT for serious cardiac, respiratory or vascular abnormalities is crucial in averting high morbidity risks and post-operative complications. Prophylactic cholecystectomies can reduce quality of life [13], and routine cholecystectomy can be overly aggressive on asymptomatic patients [14].

To conclude, SIT is a rare condition that requires greater awareness amongst the surgical field in performing procedures such as laparoscopic cholecystectomies. When left upper quadrant pain persists along with other anatomical anomalies, it is vital to hold a wide index of suspicion for SIT. This requires a holistic, multidisciplinary approach, supported by experienced surgeons capable of adapting to the technical challenges that the heterotaxical anatomy presents.
